# Factors influencing the nutritional behavior of Syrian migrants in Germany — results of a qualitative study

**DOI:** 10.1186/s12889-021-11268-9

**Published:** 2021-07-06

**Authors:** Alexandra Sauter, Salma Kikhia, Julia von Sommoggy, Julika Loss

**Affiliations:** Department for Epidemiology and Preventive Medicine, Medical Sociology, University of Regensburg, Regensburg, Germany

**Keywords:** Syria, Immigrants, Nutrition transition, Germany, Health promotion, Qualitative research, Migration, Diet

## Abstract

**Background:**

Syrian migrants represent the third-largest group of foreigners in Germany and are therefore potential users of health promotion initiatives, including nutrition programs. It is little known how (healthy) nutrition is understood and implemented by this group and which factors influence their experiences related to food and eating in the host country. Thus, this study aimed to explore the importance of (healthy) nutrition, facilitators, and barriers of a preferred diet; nutritional changes in relation to the country of origin; and how nutrition may change with increasing length of stay.

**Methods:**

Thirty semi-structured qualitative interviews with Syrian migrants (male = 16, female = 14, 18–35 years, length of stay 10–68 months) were conducted in 2018. Seventeen migrants could be followed-up after 12 months and were interviewed in 2019 again and were asked for changes in their nutritional behavior. Interviews were conducted in German, English, or Arabic, transcribed and translated into English if necessary. For analysis, an abbreviated version of the Grounded Theory was used.

**Results:**

We identified six overarching themes that described influencing factors on a favored diet in Germany over the course of stay: (1) managing everyday life; (2) intercultural contact with local residents; (3) social context of cooking and eating; (4) ambiguity toward Arabic food; (5) mistrust toward certain types of food; and (6) influence of postmigration stressors. In general, the importance of nutrition is high among Syrian migrants. However, daily stressors, the lack of practical knowledge of how to cook favored dishes, and food insecurity in the new food environment make it difficult to obtain a preferred diet. With increasing stay, many developed a higher awareness of healthy eating, mainly due to a new independence or influences from the social environment in Germany.

**Conclusion:**

Results highlight the need for health promotion interventions to be more responsive to the specific needs of Syrian migrants, including nutrition. Syrian migrants differ in their capabilities, needs, and aims, and they should be addressed differently by health professionals, social services or migrant specific services. Future research should continue to focus on the living conditions of Syrian migrants and its influence on nutrition.

## Introduction

Fostering healthy nutrition is a core public health aim. Unfavorable dietary patterns, such as diets rich in fat and sugar or an increased consumption of processed meats, are closely linked to the development of chronic diseases such as type 2 diabetes [1, 2], certain cancers (e.g., colon cancer) [3, 4], and cardiovascular diseases [5, 6]. Cardiovascular diseases such as atherosclerosis, high blood pressure, and coronary heart disease are the leading causes of death in industrialized countries [7].

According to the World Health Organization, a healthy nutrition for adults comprises of a balanced food variety consisting of fruits, vegetables, legumes, nuts and whole grains. In addition, less than 30% of daily energy intake should come from fats [8]. Unsaturated fats (e.g. contained in fish, avocado and nuts), are preferable compared to saturated fats (e.g. contained in high-fat meat, butter, coconut oil, cheese, ghee). In addition, less than 10% of daily energy intake should come from sugars added to foods or beverages or sugars naturally present in honey, syrups and fruit juices [9]. The daily salt intake should be less than 5 g [8].

Populations from the Middle East have different dietary patterns from those from North America and Europe [10]. The Middle Eastern diet includes a range of foods with often complex recipes. Due to the food variety, it is hardly possible to classify the typical Middle Eastern, or Arabic, cuisine as ‘healthy’ or ‘unhealthy’. Also, dishes and cooking styles can differ greatly between countries and country regions. Many traditional Middle Eastern dishes have been shown to have a favorable nutrient profile, containing little sodium and refined starches and being rich in vegetables [11].

Many factors influence people’s eating habits and their meanings related to food and eating, including knowledge about nutrition and skills in food preparation. Literature has shown that the surroundings and social contexts in which a person lives also impact nutritional habits [12, 13]. According to Story et al. [14], the so-called “food environment” includes the social environment, which is characterized by the influence of family and peers, the physical environment (e.g., places where people live and work and their access to food retailers and services), and the macro-level environment, including societal and cultural norms, food marketing, and political structures.

Some studies show that after migration to the host country, migrants may find it difficult to become familiar with the new food environment, which can result in food insecurity [15–18]. The available food and dietary patterns in the host country interact with a migrant’s culture, ethnicity, gender, and place of origin, impacting their dietary behavior. Over time, migrants often adopt new eating behaviors in their host country while they acculturate to their new environment [19].

A number of studies — predominantly from the United States and Australia — have shown that refugees and migrants from various countries are indeed challenged by facing a foreign food culture, which often leads to unhealthy food choices or a so-called westernization of food habits. This includes the consumption of prepared and fast food, which is high in calories and low in nutritional value [20–23]. For example, Kh Al-Farhan et al. [24] showed that Arabic students, after living in the United States for more than 6 months, tend to develop an eating behavior that is less traditional and more westernized. Sastre and Haldeman [25] describe a rapid dietary acculturation among adolescent refugees migrated to North Carolina, with an increase in consumed sugary and fast food.

The group of Syrian migrants has gained significant relevance in Germany in recent years. The civil war in Syria, which started in 2011, has been responsible for one of the largest refugee crises in modern history (6.3 million people fled the country in 2017). Germany became the main destination for Syrian migrants in Europe. There are around 700,000 Syrian migrants in Germany, making them the largest group of registered asylum seekers and the third-largest group of foreigners in Germany [26].

In Germany, the period of applying for asylum is associated with several changes of residence. After arrival in Germany, asylum seekers live in an initial reception facility or arrival center for up to 6 months, or until their application is decided on [27]. During this period, they can also be allocated to another facility, for instance for family reunification. The reception facility is responsible for providing food [28]. After 6 months, the obligation to live in the initial reception center ends and asylum seekers can move to a collective accommodation facility or apartment [28]. Collective accommodations are often smaller and spread all throughout the federal states. Persons who are entitled to asylum receive a residence permit for one up to 3 years and are free to choose their place of residence in Germany [28]. German law does not set a time limit for the Federal Office for Migration and Refugees to decide on an application. In 2019, procedures took 5.1 months on average for Syrian applicants [29]. Additionally, it is possible to enter the country via visa, for example to study or work. In this case a person must be able to support oneself and to finance private housing [30].

Still, little is known about the concepts of health, well-being, and health-related lifestyles, such as diets and physical activity, that prevail among this population. So far, German studies on Syrian migrants have mainly focused on their physical health upon arrival in Germany [31, 32], their mental health [33–35] and general health behavior among Syrian subgroups [36], and the legal situation regarding health insurance in Germany [37]. The focus of these studies was not always exclusively on Syrian migrants but included other new arrivals.

This study aims to lend a better understanding of the experiences related to food and eating of Syrian migrants and of the barriers and facilitating factors relevant for (healthy) nutrition that occur in their everyday lives. It addresses the following questions:

1. How relevant is a healthy diet in the context of migrating to and settling in Germany?
2. How do experiences related to food and eating change during the stay in Germany over time, as compared to the life in Syria?
3. What are the enablers and barriers to maintaining the preferred diet in Germany?

## Materials and methods

### Design

We conducted a longitudinal qualitative study using semi-structured in-depth interviews at two different times (t_0_ and t_1_ after 12 months). No previous study has reviewed the nutritional behavior of Syrian migrants living in Germany yet. Therefore, we chose an explorative research design, as it allows people to fully grasp the perspectives of the participants, to probe into interesting aspects, and gain rich data that help to make causal links between contexts and behavior [38]. The longitudinal design [39, 40] helps track changes in nutritional behavior and influencing factors over time [41], as the situation of newly arrived migrants may undergo dramatic changes within the first few years of their stay, e.g. changes in residence status, work permission or family reunification.

The study was performed in accordance with the Declaration of Helsinki. Study protocol, information sheet, and consent form received ethical approval from the Ethics Committee of the University of Regensburg (18–1024-101).

### Sampling and participant recruitment

We included Syrians who had applied for asylum and were living in Germany or had entered the country with a visa. We decided to include both groups in the study as it was possible for interviewees to buy their own food and to cook independently. This is also true for those living in collective accommodation facilities. We excluded people who lived in an initial reception facility where food is provided by the facility.

#### Interviews t_0_

We followed a purposive sampling strategy to include people with (a) different gender, (b) different educational backgrounds (education between 6 and 12 years), and (c) different length of stay (less than 1 year, up to 5 years, to ensure participants had settled in their new environment).

The participants were recruited in a city (150,000 inhabitants) in southern Germany through various channels:

- Sending e-mails to different local associations working with migrants
- Visiting language courses and “women get together groups” to address participants personally
- Posting requests on Facebook groups of the local Arabic community
- Pinning notes on bulletin boards on campus

Participants received an information document (in Arabic) explaining the purpose and content of the study. If needed, additional oral explanation was provided. Every participant signed an informed consent form for the interview, the audio recording and the scientific use of their accounts, and a form giving written permission to be contacted again after 12 months.

At t_0_, we conducted interviews with 30 Syrians (14 female) aged 18 to 35 years. The recruiting of interviewees continued until saturation, the point in the ongoing analysis when no new themes appeared [42]. Therefore, after each interview, both interviewers (A.S., S.K.) discussed its content. S.K., being a Syrian native, sometimes acted as cultural mediator (explaining cultural characteristics in the responses). The discussions helped to capture what new information was elicited during an interview and what topics were already known from other interviews.

#### Follow-up interviews t_1_

All interviewees were re-contacted after 12 months via e-mail. When there was no response, other channels were used (e.g., telephone calls, short messages, and social media accounts). Twenty-two participants were successfully contacted again, of whom 17 agreed to participate in follow-up interviews. The other five had no interest in a further interview.

### Materials

#### Interviews t_0_

We used the Theoretical Domains Framework (TDF) (Michie et al., 2005) to develop the questions for the topic guide. The TDF is a validated model consisting of 84 constructs from various psychological models of behavioral change, which allows a broad and detailed operationalization of the influencing factors of behavior [43, 44]. Additionally, we used specific literature on migrants’ nutritional behavior to specify questions [21, 22, 45]. Table 1 shows a generalized version of the topic guide, including prompt questions that stimulate more specific answers. We also used a short questionnaire to collect socio-demographic data and general information on the interviewees’ residence in Germany.

**Table 1:** Topic guide for semi-structured interviews t_0_

TDF-Domain	Main questions
	***1. What is a healthy diet for you?***
*Knowledge,* *Skills*	***2. Where have you learned about healthy eating?***
*Nature of behavior,* *Importance*	***3. How important is it for you to eat healthily?*** ➢ If so: *What are you doing to eat healthily?* ➢ If not: *Why isn’t it important to you?*
*Beliefs about consequences*	***4. In what ways do you think nutrition can affect your health?***
*Beliefs about capabilities,* *Environmental context and resources,* *Social influence,* *Social role,* *Motivation and goals,* *Skills*	***5. What factors impact your eating behavior?*** ➢ *What influence do family members and friends have?* ➢ *Do you have certain rituals or customs concerning your eating behavior?* ➢ *Is your culture or religion affecting your eating behavior?* ➢ *Do you know how to prepare the desired dishes?*
*Beliefs about capabilities,* *Environmental context and resources,* *Skills*	***6. In which way have your eating habits changed since you moved to Germany?*** ➢ *How satisfied are you with what you eat in Germany?* ➢ *Do you eat differently now than in Syria? Why?* ➢ *Can you buy the desired food in your neighborhood?* ➢ *Are there any “new food items” that you do not know from your home country? ➔ Do you integrate these products into your diet?*
*Motivation and goals,* *Environmental context and resources*	***7. What would help you eat the way you would like to?***

#### Follow-up interviews t_1_

The topic guide for the follow-up interviews were primarily based on the questions of the t_0_ topic guide, but additional individual questions were asked, depending on the course of the interview and the specific situation of the interviewee. Also, the short questionnaire from t_0_ was used again.

### Procedure t_0_ and t_1_

The interviews were led by two authors (A.S., S.K.) 06–07/2018 (t_0_) and 09/2019–01/2020 (t_1_). The participants decided in which language (German, English, or Arabic) the interview was to be conducted. One researcher (S.K.) conducted interviews in the first language of the participants (t_0_: *n* = 18, t_1_: *n* = 7), A.S. conducted the interviews in German or English (t_0_: *n* = 12, t_1_: *n* = 10). Interviews were conducted at a place convenient for the participants (e.g., at their homes, in the venue of the language course, at the researchers’ office). All participants completed the short questionnaires at both times (see Table 2). All interviewees received 30 Euros as incentive after each interview.

**Table 2:** Characteristics of interviewed participants t_0_ and t_1_

	Pupil/ Student/Trainee		Worker		At home (unemployed/housewife/waiting for training)		Total	
	t_**0**_ *(n = 10)*	t_**1**_^a^ *(n = 13)*	t_**0**_ *(n = 5)*	t_**1**_ *(n = 1)*	t_**0**_ *(n = 15)*	t_**1**_ *(n = 3)*	t_**0**_ *(n = 30)*	t_**1**_ *(n = 17)*
Mean age	24	24, 92	26, 8	29	25, 53	22, 3	25, 3	24, 7
Sex	m = 9, f = 1	m = 10, f = 3	m = 3, f = 2	m = 1	m = 4 f = 11	m = 1, f = 2	m = 16, f = 14	m = 12, f = 5
Residential status:								
*Permanent*	1	–	–	–	2	–	3	1
*Temporary*	9	12	4	1	11	3	24	15
*Waiting for approval*	–	1	1	–	2	–	3	1
Months since arrival in Germany:	∅ 37 (19–68)	∅ 49, 23 (30–83)	∅ 29 (15–46)	64	∅ 28, 93 (10–55)	∅ 45 (37–52)	∅ 31, 6 (10–68)	∅ 49, 35 (30–83)
German language level^b^								
*Fluent (C2-C1)*	5	9	2	1	4	1	11	11
*Advanced (B2-B1)*	5	4	2	–	2	1	9	5
*Beginner (A2-A1)*	–	–	1	–	7	1	8	1
Marital status:								
*Single*	9	13	3	1	6	2	19	16
*Married*	1	–	2	–	8	1	11	1
School education:								
*0–9 Years*	–	–	4	–	6	1	12	1
*10–12 Years*	10	13	1	1	8	2	18	16
Residential area Syria^c^								
*Medium city (20.000–99.999 inhab.)*	5	4	1	1	8	2	14	7
*Big city (from 100.000 inhab.)*	2	4	–	–	3	–	5	4
*Metropolis (from 1 mio. Inhab.)*	1	3	4	–	3	1	3	4

^a^ The status of single persons has changed by starting apprenticeships or studies

^b^ Two participants did not provide information about their German language level

^c^ Three participants did not provide information about their hometown in Syria

### Analyses

All interviews were transcribed verbatim in the original language. Arabic transcripts were translated into English by S.K. or a professional translation office. The final transcripts were de-identified and continuously numbered (IP01-IP30). The analysis was based on Glaser and Straus’s “Grounded Theory” approach [46], following the recommendations for the pragmatic application of an “abbreviated grounded theory” [47]. The following steps of the Grounded Theory were considered:

#### Interviews t_0_

First, interviews were conducted until saturation. Second, transcripts were read and re-read several times, and open coding was undertaken by two authors (A.S., J.v.S.) independently to identify the phenomena occurring in the data and to formulate first summarizing categories. Results were discussed and adapted. Third, axial coding was undertaken by A.S. to outline key categories. Focus was also placed on identifying contradictory data. To ensure rigor of the analytical process [48, 49], all identified key categories were discussed with a third author (J.L.) before patterns and relationships between categories were identified and higher order themes were defined.

#### Follow-up interviews t_1_

In a first coding round, recurring categories from the t_0_ interviews and new categories in the t_1_ interviews were identified. In a second coding round, the relationships between new categories from t_1_ and categories from t_0_ were analyzed. Similarities and differences between the categories were identified. Findings were discussed with another author (J.L.). Finally, higher order themes defined in t_0_ were expanded in terms of the newly identified categories from t_1_.

## Results

We identified six main themes describing the factors influencing a preferred nutritional behavior in Germany: (1) managing everyday life; (2) intercultural contact with local residents; (3) social context of cooking and eating; (4) ambiguity toward Arabic food; (5) mistrust toward certain types of food; and (6) influence of postmigration stressors.

### Shopping and eating as part of managing everyday life in Germany

Many interviewees migrated to Germany without family members. Interviewees in the t_0_ interviews often described that while living in Syria, they had not given the issue of (healthy) eating much consideration. Having lived in their parents’ houses where they had relied on their mothers for food preparation was named as a main reason why interviewees had not dealt with nutrition issues yet. Migrating to Germany caused a dual biographical disruption: First, the interviewees had moved out of their parents’ homes and had been forced to organize their everyday lives and households on their own (including food shopping and cooking). Second, migrating to a foreign country with a different language and dealing with the authorities, culture, and behavioral norms made coping with everyday life even more difficult.

*“Back there (in Syria) my mother cooked, and the food was ready. Here I have to do the efforts to cook. Some stuff I’m not even able to prepare. It is too much work to cook for oneself. Yes, this has changed” (IP13, m., t*_*0*_*).*

Interviewees in t_0_ described different ways to handle their new living situation. Male and female narratives especially differed from each other in this regard.

For (married) women, the preparation of food often seems to belong to their perceived role within the family. Some female interviewees had already been married in Syria and maintained their family obligation of grocery shopping and cooking after migration. For the majority of the male interviewees, cooking is more of a challenge, according to their reports. Some show no interest in learning cooking skills at all and state that they are satisfied with premade and fast food, as long as the daily energy supply is covered. Some male interviewees referred to a future stage in their life when their (potential) partner could take over the task of cooking (healthy) meals.

*“Satisfied, but also not satisfied [with the current nutrition]. I have no options. There’s nothing that I could do to change it (…) But something that will make me eat more healthily is when my wife arrives. She's on her way [to Germany]” (IP10, m, t*_*0*_*).*

Conversely, some migrants perceive the newly acquired responsibility to do the shopping and food preparation themselves as an opportunity to plan their diets according to their own wishes. Some start to question the previously known diet of the parental home and emancipate themselves by looking at the nutrition facts and origins of the products used. They use this knowledge to change their eating habits to be in line with their own preferences (e.g., using less fat and meat, using “healthier” oils).

*“[My nutritional behavior] changed a lot. In Syria I never had to cook or worry about food, but here I have to decide on my own what to cook, and do my groceries-shopping accordingly. Furthermore, I have to think about what is best and cheapest to buy. Shopping for groceries became healthier (…) because now I do it myself and decide for myself what to buy (…) [I] actively look at the source of every product and whether it’s organic or not” (IP09, m., t*_*0*_*).*

In the follow-up interviews, some participants reported an active network of the Arab community in Europe on the Internet. Some referred to migrant bloggers who were reported to host their own food-related social media accounts, explaining how to prepare Arabic dishes and which European products may be substituted for traditional Syrian ingredients. This online guidance helped them to improve their cooking skills and to prepare food in a more preferred way.

*“I have no experience how to cook. But there are many refugees who live in Europe who have their own YouTube channels. They explain how to prepare Syrian dishes with ingredients available in Europe. (…) For example, there are differences between meat in our country and meat in Europe. What are the alternatives [in Europe]. And that really helps”* (IP05, m., t_1_).

A few people, mainly women and higher educational level, also reported that they now try to reinterpret the traditional Arabic cuisine to be prepared in a healthier way.

*“I try to make those traditional recipes, without meat, with alternatives (…) If we want to eat bulgur or something, I try vegetarian bulgur, and it tastes good”* (IP03, f., t_1_).

Apart from gender differences, in both interviews t_0_ and t_1_, there are differences between occupational states. Students, apprentices, and employees found it more challenging to grocery shop and cook fresh meals daily, as compared to those interviewees who stay at home or are enrolled in a language course. For the interviewees who were studying or working, the language difficulties led to a daily additional time burden.

*“My lifestyle in Syria was very healthy. But that’s because of a lot of factors like having my family there, and also having time. In Germany my lifestyle has changed to become totally unhealthy. I’m always at the university trying to study. So, I always forget to eat, to the extent that I don’t feel hungry anymore. I have been losing weight all the time — 10 kilos so far” (IP06, f., t*_*0*_*).*

### Intercultural contact with local residents’ influences, eating behavior, and preferences

Especially in the beginning of the residency, changes in diets did not come easy for the newly arrived migrants, even when they were critical of certain aspects of their traditional food.

*“It's hard for us to accept food items other than those we're used to. I have had these eating habits for 24 years. In general, our [Syrian] food is unhealthy, with lots of fat and meat. But we cannot change our habits quickly. There are also Arab shops where we still buy our food. Nothing has changed for us” (IP21, f., t*_*0*_*).*

But with ongoing stay, the observation of the residents’ behavior from the host country (e.g., customers in supermarkets or restaurants) induced the interviewees to reflect more upon the conventions prevalent in the host country, according to the interviews. The majority of respondents perceived the shopping and eating habits of local residents to be health-conscious and disciplined compared to the traditional Arabic cuisine. Some interviewees reported that, after several months of residence, they started to question their traditional behavior or even changed their diets to be healthier, feeling that a health-promoting behavior would be sort of a norm in Germany.

*“Living among Germans, I noticed that their health is one of the most important things for them. When we go to a restaurant together, they often order vegetarian or light meals (…) They pay a lot of attention to their health (…) I appreciate the Germans' view of these things a lot. Through the Germans, I have really started to reflect on my diet” (IP16, m. t*_*1*_*).*

### Social context of cooking and eating within the household structure influences diet

But not only the observation of behavior has an impact on what interviewees perceive as healthy or unhealthy food, also their social and living environment seems to have a significantly influences how they cook and what they. In this context, the household structures appear to have an impact on one’s diet. Joint cooking evenings with (German) flat mates were described as opportunities to talk about nutritional knowledge, specific practices, beliefs, and values regarding food preparation.

When there is no time to buy food or cook, interviewees often describe that they rely on the cooked food of their roommates, even if this food is not always their idea of an appropriate healthy meal.

*“My friend [flat mate] likes to eat things that are fried. So most of the days he makes French fries or something like this. I really gave up on this kind of food, frying the food (…) but on a lot of days I don't have time, so I come home, heat any food that I find and eat it because I’m hungry. If it comes to me, I would eat better things. For us, as Arabs, if you live together, then you have to eat together, to an extent at least”* (IP14, m., t_0_).

Changes in household structure are a particularly relevant aspect in the lives of newly arrived migrants, as they move frequently in the beginning (e.g., moving out from asylum camps, moving into temporarily rented rooms until an apartment or shared room is found). Over time, the interviewed migrants relocated in for family reunification, start of employment, or enrollment in a study program. After a move, a person’s social context may change, and with it, the external influences on his or her eating habits, which was often reported in the t_1_ interviews.

*“Two months ago, I moved back [from my single apartment] to a shared flat [with Germans] because it had been a bit lonely (…) and my eating habits had become very bad. I had less contact with Germans and I invited Arabic friends, (…) every time we met, we cooked something from our culture. It was always rich in calories. I gained weight and I wasn't taking care of my health anymore. Since I moved back to a shared flat, my behavior has changed again (…) I wanted to be among Germans again so that I don’t live in a parallel society. My flat mate is very concerned about his nutritional behavior, and we discuss food topics all the time. That’s why my eating habits have improved once again”* (IP01. m., t_1_).

### Ambiguity toward Arabic food impedes food decisions

Describing the Syrian food and eating culture, the interviewees always pointed out that Syrian people do not worry about healthy nutrition and that taste and enjoyment of food always takes priority over a health aspect. The traditional Arabic cuisine was rated as unhealthy, which they mainly attribute to the fat and oil content and the large quantity.

*“Because, as Arabs, or as Syrians, we are not careful with what we eat. We don't watch out for how much fat food contains, we simply eat it”. (IP16, m., t*_*0*_*)*

Also, joint Syrian meals with peers from the same home country were described as an important social event, which could even mitigate homesickness. Thus, although interviewees expressed the relevance of a healthy and balanced diet, many do not manage to adjust their daily diet accordingly, regardless of their level of education.

*“I had no problems back then (in Syria). I was there with my family. And therefore, the taste (of Arabic food) brings back this memory, brings it back somehow”* (IP08, m. t_1_).

The longer the stay, the more interview partners reported they were torn between their desire to find consolation in enjoying Arabic meals and their rational concern about the anticipated risks this may pose for their health. As a consequence, they experience a permanent ambiguity toward Arabic dishes, struggling to find a compromise in everyday life or to justify their behavior.

*“I could cook it (the Arabic recipes) in a healthier manner, but it wouldn’t taste the same way that I’m used to. That can be seen as a barrier. Something that I have been used to since I’m very little, it’s impossible that I change it” (IP12, m., t*_*0*_*).*

### Distrust toward certain types of food influences food choices

Especially in the t_0_ interviews, interviewees explained that they distrusted certain food items in German supermarkets, such as frozen vegetables or meat, even if it is labeled to be halal (permissible, adherent to Islamic law). People also reported being afraid of forbidden substances such as alcohol or pork hidden in products. Others were discontent with the food quality (e.g., referring to vegetables). Especially people with low education and little German language level, reported of discomfort shopping in German supermarkets, with the consequence of having a perceived unhealthier diet.

*"We would like to change our diet, but we only buy from Arabic shops because we don’t speak German (…) I know that German supermarkets offer better food, but I am afraid of forbidden substances like pork or alcohol (...) and I stick to my usual unhealthy eating habits." (IP22, f., t*_*0*_*)*

Some interviewees described how they distrust dishes prepared by others, such as when eating out (e.g., in a restaurant or at dinner parties).

*“I don’t eat anything that has pork or alcohol in it. Once, a friend invited me. She offered me food and said there was no pork in it. But when I tasted it I felt that it did contain pork, but she assured me that it didn’t. So I ate it and then found out that it was pork. It was a tricky situation” (IP07, m., t*_*0*_*).*

In the follow-up interviews, many participants (with higher educational level) reported they changed their views regarding products of the Arabic shops. The quality of the halal meat was rated as poor, with the consequence of buying in local German supermarkets, which seems to be an improvement in their diet for some.

*“We are here in a foreign country and have to adapt to the local conditions, because halal meat is not always available. The meat in the German market is partly even better than the meat in the Arabic shops. In the end you can’t be sure if that is really halal” (IP16, m, t*_*1*_*).*

### Postmigration stressors (indirectly) influence eating behavior

During the process of migration and the first months after arrival, regardless of the educational level, health issues were given little to no priority. The interviewees in t_0_ described that, after arriving in Germany, the highest priority was given to visiting the authorities, reunifying with family, attending language courses, and finding out whether training/working opportunities were available.

*“When we first arrived, we completely forgot about healthy food or how to take care of our health. Our priorities were to learn how to live and learn what is the most efficient way to reach our goals. What was taking all my time was: my studies, my family, my work (…) the health topic is set aside right now (…) It’s about setting priorities”* (IP16, m., t_0_).

After several months, when daily stressors decreased and migrants were able to successfully adapt to the new cultural context, find a place of housing, and improve language skills, some interviewees reported a reorientation regarding their health. Interviewees described that their perceived future prospects in the new country were an incentive to invest more in their own health, for example, a balanced diet.

*“When I was in Syria I really didn’t care about [a healthy lifestyle]. Life ends anyway. But here I started to care, because I feel that I need to be living a healthy life (…) because I want to do something in the future, for myself and for my family. So life became important” (IP12, m., t*_*0*_*).*

Follow-up interviews also show that mental health and well-being may deteriorate over time. Distress and mental strain may continue from harassing news from the home country, worries about family members, or unpleasant situations in everyday life. This could indirectly affect health behavior negatively, such as an unhealthy diet. Some named shisha smoking or eating high-calorie food (from the home country) as strategies to relieve anxiety. Others reported losing their appetites and found it difficult to eat regularly.

*“But sometimes, when I’m not feeling well, or when I’m depressed about the daily news (in Syria), I don’t care about my diet anymore. I eat everything I can find and lots of sugar. There are factors like the news, the wellbeing of my family, sometimes racism I experience. To deal with that, I sometimes eat more . . . simply to forget or turn off your thoughts”* (IP01, m., t_1_).

## Discussion

### Principal findings

The interviews indicate that for Syrian migrants in Germany, the new environment and living situation pose many barriers to eating in the desired way, but they also offer opportunities and support to improve dietary behavior. Different post-migration stressors influence eating behavior, according to the participants’ narratives. Even after the first bureaucratic hurdles are jumped and the housing situation is settled, other occupations (especially learning a new language in addition to studying or working) are frequently described as so time-consuming that little to no time is left for shopping or cooking. Consequently, some interviewees resort to eating fast food or left-overs of co-residents; others eat only infrequently. Particularly young single men, who had been regularly fed at home by their families in Syria, suddenly are left to fend for themselves and are often overwhelmed with the task of preparing a proper meal. Therefore, social support (e.g. by co-residents or family members) plays a very important role.

Some interviewees — predominantly students — often interact with German residents and strive to adjust to their way of preparing meals, which is perceived to be very healthy. Others distrust German food items and/or food labeling in supermarkets and prefer to buy groceries only in Arab shops, even after years in the host country. Opinions on Arabic food were contradictory: While interviewees repeatedly stressed that they considered Arabic meals unhealthy (mainly because of their fat content), the (joint) consumption of traditional Middle Eastern dishes played a major role in their lives, making them feel connected with their homeland. This tension seemed to trouble the participants, as they felt obliged to eat healthier now that they were staying in Germany, facing better long-term prospects and a perceived social norm of healthy living — but they still craved the comfort of traditional dishes.

The follow-up interviews showed that some of the factors influencing the Syrian migrants’ eating behavior could change over time, such as when they moved on to a different housing situation, were re-united with (or separated from) family members, or when the nature of their employment status changed. However, these changes were rather accidental and could hardly be attributed to systematic developments over time in the lives of Syrian migrants in Germany.

### Strengths and limitations

We were able to recruit a substantial and very heterogeneous sample of Syrian migrants, based on their age, length of stay, language level, residence permit, marital status, and education and inheritance status. We also succeeded in collecting data among Syrian migrants who lived secluded lives and were not well educated — mainly women — by a comprehensive recruiting system with many community partners. Furthermore, we could conduct a second interview after a year’s time with more than 50% of the participants, thereby tracing how the change in social and environmental contexts impacts eating behavior.

A limitation is the selection bias in the t_1_ sample. Specific groups were lost to follow-up, predominantly married women with low education, who represent a particularly vulnerable group. Overall, the t_1_ sample consists of a more privileged group of mainly male migrants, with a higher level of education, better language skills, and being a student or trainee. Migrants living in refugee accommodations are not represented in the t_1_ sample.

The time span of 1 year may also be too short to grasp developments and dynamics in the post-migration life course of Syrian migrants. For future studies, it may be reasonable to have a more homogenous t_0_ sample referring the length of stay (e.g. maximum of 12 months) and to have two follow-up times after 12 months each, in order to be able to map a clearer time course. Still, we found that social and physical changes also contributed to changes in eating behavior, highlighting the importance of food environment.

The study design allowed interviewees to choose the language of the interview, which has given them more confidence in expressing themselves during the interview. A potential limitation lies in the translation process. We cannot fully exclude discrepancies between the narratives in the original language (especially spoken “informal” Arabic) and their transcription and translation to English.

There may have also been an interviewer effect. Some interviews may include biased statements due to the gender of the two interviewers (both females). Likewise, social desirability may be a problem in some of the answers, especially considering how often the interviewees emphasized the relevance of health-promoting behaviors and healthy nutrition. Migrants may have felt they needed to be polite to interviewers and show their endeavor to integrate with, or comply with, German culture. The interviewers tried to counteract this phenomena by repeatedly pointing out that there were no “right” or “wrong” answers, and the study was about understanding, not judging, the migrants’ situation.

For example, it is noticeable that the interviewees described the German culture as very health-conscious, especially when it comes to a healthy diet, but rated their own culture, especially Syrian food, as fatty and unhealthy. Comparing this perception with current epidemiological studies from Germany, a clear discrepancy between the migrants’ impressions and the actual situation becomes apparent. Studies show that the majority of the German population does not manage to comply with the national and international recommendations on daily food intake. Only 5% of women and 7% of men aged 18 to 79 achieve the recommended portions of fruit and vegetables per day [50, 51]. However, data also show that the prevalence of vegetarians is highest in the group of 18–34-year adults with higher socioeconomic status [52]. This could be a possible explanation for the biased perceptions of migrant respondents, as many interviewees lived in an academic characterized living or working context (attending college, living in student housing). Also, charity organizations often cooperate with universities and student volunteers. Overall, it should be noted that the interviewees rated themselves significantly worse in their dietary behavior than is actually the case in comparison to the society of the host country, where 67% of men and 53% of women are overweight [53].

### Comparison with other studies

To our knowledge, this is the first study to investigate factors influencing the nutritional behavior of Syrian migrants in Germany. Few studies had explored health and health behavior of Syrian migrants.

A Brazilian research project named “The food as a refuge” used ethnography and semi-structured interviews to explore the food culture of Syrian migrants in São Paulo [54, 55]. The participants preferred their previous eating habits over those of the host country, which were considered boring. In line with our findings, the Brazilian migrants reported that preparing and consuming traditional Arabic dishes created a feeling of home. They would also not accept Arabic food that was not cooked by Arabs themselves. This topic does not explicitly appear in our interviews but is reverberated in the described distrust of dishes prepared by others.

Some of our results are also confirmed in studies in other ethnic groups. Hunter Adams [56] described how Congolese, Somalis, and Zimbabweans living in Cape Town, South Africa, “romanticized” food items of the home country. These products were considered fresher and healthier than products of the host country, as produce could be bought directly from the growers, and no additives were used.

Similarly, a qualitative study [21] with Cambodian, Somali, Mexican, und Sudanese migrants in the United States emphasized the emotional role of traditional food of the home country. Participants were not willing to adopt food practices of the host country although they described their own traditional cuisine as fatty; distrust toward supermarket products was reported.

### Implications for policy and practice

Lack of trust and knowledge of food in the host country can lead to poor food choices. Daily stressors and post-migration stressors can also act as barriers to a preferred diet. Many of the migrants, however, have a strong interest in nutrition and in establishing a diet that they consider healthy.

But health promotion and health education targeting migrants’ eating habits also need to consider that nutrition is strongly linked to the migrants’ origin and identity, and eating fatty Arabic food may be not only culturally important but also a means to consolation and comfort in a disconcerting new life situation. Therefore, culturally sensitive programs are needed that help (young) migrants to learn about the foreign culture but also to empower them to find their own ways to have balance between the different cultures without having the feeling of losing part of their own identity.

Low threshold programs with peers from the same origin or bilingual health experts, as described by Lecerof et al. [57] and Renzaho et al. [58], can help to inform recently settled migrants in their native language about their new food environment, the cultivation and processing of German food, food standards, and hygiene regulations in German supermarkets. Based on our results, it also seems reasonable to start supermarket initiatives where Syrian migrants and supermarket operators jointly inspect products and discuss the need for an extended labeling regarding alcoholic or halal ingredients or the origin and growing of fruits and vegetables (e.g., greenhouse or open-field grown). This could also benefit migrants from other Muslim countries.

Our study also highlights the importance of promoting personal resilience to cope with war and migration. Riedel [59] acknowledges that programs for newly arrived migrants must focus on people’s resistance resources and salutogenetic capacities to deal with acculturation stressors or traumatic experiences. This can be done by requiring low-threshold psychotherapeutic offers in the Arabic language, such as the HELP@APP project [60].

Diets are not only influenced by individual factors, such as knowledge about healthy food, but also by the surrounding environment (e.g., housing conditions, available grocery supply, access to the labor market, or educational services). The interviewees’ reports of flat sharing with German residents were encouraging: In these circumstances, Syrian migrants did not only perceive improving their language but also reflecting upon their eating habits and being motivated to try new dishes. To promote the bi-directional exchange of knowledge and experiences between migrants and local people, training companies or universities could also be involved in food programs. At joint lunches or cooking evenings, different ways of food preparation can be exchanged which may be a rewarding experience for both sides, as the Syrian cuisine offers a diverse range of vegetable-based dishes that can enrich the diet of local residents.

Furthermore, findings outlined the need for community-based efforts to provide relevant bilingual information regarding life in the new community (e.g., contact persons, addresses for childcare centers, tips for applying for financial support, information about the health system). There are primary initiatives, such as the Integreat App (https://integreat-app.de/en), which enables stakeholders and policymakers to provide relevant information of their community for migrants in a free bilingual app. Such low-threshold access to information can be a great relief, especially for newly arrived migrants.

## Conclusion

For some Syrian migrants, the new start in Germany is an opportunity to deal more consciously with the topic of food and nutrition and to find new ideas about how to feed themselves in exchange with German culture. Conversely, Syrian migrants also face many challenges when they strive to maintain their favored diet in the new living environment. Also, food and health are not always a high priority in the beginning of residence as biographical ruptures, homesickness, additional burdens due to learning a new language and coping with bureaucracy, and a loss of social contacts impede daily life. Thus, public-health efforts need to be more responsive to the specific needs of Syrian migrants, including nutrition. Syrian migrants are a heterogenous group which differ in their capabilities, needs, and aims which should be addressed differently by health professionals, social services or migrant specific services. Future research should continue to focus on the living conditions of Syrian migrants and its influence on nutrition. The specific needs of various sub-groups (e.g., mothers, single men) must also be considered in future research.

## Data Availability

The qualitative data material used during the current study are available from the corresponding author on reasonable request.

## References

[1] Salas-Salvado J, Martinez-Gonzalez MA, Bullo M, Ros E (2011). The role of diet in the prevention of type 2 diabetes. Nutr Metab Cardiovasc Dis.

[2] Ley SH, Hamdy O, Mohan V, Hu FB (2014). Prevention and management of type 2 diabetes: dietary components and nutritional strategies. Lancet.

[3] Azrad M, Blair CK, Rock CL, Sedjo RL, Wolin KY, Demark-Wahnefried W (2019). Adult weight gain accelerates the onset of breast cancer. Breast Cancer Res Treat.

[4] Albuquerque RC, Baltar VT, Marchioni DM (2014). Breast cancer and dietary patterns: a systematic review. Nutr Rev.

[5] Dieter BP, Tuttle KR (2017). Dietary strategies for cardiovascular health. Trends Cardiovasc Med.

[6] Bowen KJ, Sullivan VK, Kris-Etherton PM, Petersen KS (2018). Nutrition and cardiovascular disease-an update. Curr Atheroscler Rep.

[7] de Jesus JM, Kahan S, Eckel RH (2016). Nutrition interventions for cardiovascular disease. Med Clin North Am.

[8] World Health Organization. Healthy diet. WHO; 2020. Available from: https://www.who.int/news-room/fact-sheets/detail/healthy-diet. Accessed 20 Nov 2020.

[9] World Health Organization (2015). Guideline: sugar intake for adults and children.

[10] Hashemian M, Farvid MS, Poustchi H, Murphy G, Etemadi A, Hekmatdoost A, Kamangar F, Sheikh M, Pourshams A, Sepanlou SG, Fazeltabar Malekshah A, Khoshnia M, Gharavi A, Brennan PJ, Boffetta P, Dawsey SM, Reedy J, Subar AF, Abnet CC, Malekzadeh R (2019). The application of six dietary scores to a middle eastern population: a comparative analysis of mortality in a prospective study. Eur J Epidemiol.

[11] Issa C, Salameh P, Batal M, Vieux F, Lairon D, Darmon N (2009). The nutrient profile of traditional Lebanese composite dishes: comparison with composite dishes consumed in France. Int J Food Sci Nutr.

[12] Pitt E, Gallegos D, Comans T, Cameron C, Thornton L (2017). Exploring the influence of local food environments on food behaviours: a systematic review of qualitative literature. Public Health Nutr.

[13] Glanz K, Sallis JF, Saelens BE, Frank LD (2005). Healthy nutrition environments: concepts and measures. Am J Health Promot.

[14] Story M, Kaphingst KM, Robinson-O'Brien R, Glanz K (2008). Creating healthy food and eating environments: policy and environmental approaches. Annu Rev Public Health.

[15] Osei-Kwasi HA, Nicolaou M, Powell K, Terragni L, Maes L, Stronks K (2016). Systematic mapping review of the factors influencing dietary behaviour in ethnic minority groups living in Europe: a DEDIPAC study. Int J Behav Nutr Phys Act.

[16] Terragni L, Garnweidner LM, Pettersen KS, Mosdol A (2014). Migration as a turning point in food habits: the early phase of dietary acculturation among women from south Asian, African, and middle eastern countries living in Norway. Ecol Food Nutr.

[17] Hadley C, Patil CL, Nahayo D (2010). Difficulty in the food environment and the experience of food insecurity among refugees resettled in the United States. Ecol Food Nutr..

[18] Khakpour M, Iqbal R, GhulamHussain N, Engler-Stringer R, Koc M, Garcea J, Farag M, Henry C, Vatanparast H (2019). Facilitators and barriers toward food security of afghan refugees residing in Karachi, Pakistan. Ecol Food Nutr.

[19] dela Cruz FA, Lao BT, Heinlein C (2013). Level of acculturation, food intake, dietary changes, and health status of first-generation Filipino Americans in Southern California. J Am Assoc Nurs Pract.

[20] Rondinelli AJ, Morris MD, Rodwell TC, Moser KS, Paida P, Popper ST, Brouwer KC (2011). Under- and over-nutrition among refugees in San Diego County, California. J Immigr Minor Health.

[21] Tiedje K, Wieland ML, Meiers S (2014). J., Mohamed AA, al. e. a focus group study of healthy eating knowledge, practices, and barriers among adult and adolescent immigrants and refugees in the United States. Int J Behav Nutr Phys Act.

[22] Wang Y, Min J, Harris K, Khuri J, Anderson LM (2016). A systematic examination of food intake and adaptation to the food environment by refugees settled in the United States. Adv Nutr.

[23] Delavari M, Sonderlund AL, Swinburn B, Mellor D, Renzaho A (2013). Acculturation and obesity among migrant populations in high income countries – a systematic review. BMC Public Health.

[24] Kh Al-Farhan A, Kemnic T, Becker T, Caine-Bish N, Burzminski N, Gordon K (2018). Changes in dietary behavior of Arab international students in the US. Food Sci Nutr.

[25] Sastre L, Haldeman L (2015). Environmental, nutrition and health issues in a US refugee resettlement community. Int J Cuban Health Med.

[26] Worbs S, Rother N, Kreienbrink A, Hochman O, Weick S (2019). Syrische Migranten in Deutschland als bedeutsame neue Bevölkerungsgruppe [Syrian migrants in Germany as a new and important population group]. Befunde aus der Migrationsforschung [Results from migration studies]. 61: Gesis. Leipniz-Institut für Sozialwissenschaften.

[27] UN Refugee Agency. Rights and duties during the asylum procedure. Living and accomodation. UNHCR; 2021. Available from: https://help.unhcr.org/germany/rights-and-duties/rights-and-duties-during-the-asylum-procedure/. Accessed 20 May 2021.

[28] Federal Office for Migration and Refugees. The stages of the asylum procedure. BAMF; 2021. Available from: https://www.bamf.de/EN/Themen/AsylFluechtlingsschutz/AblaufAsylverfahrens/ablaufasylverfahrens-node.html. Accessed 20 May 2021.

[29] Informationsverbund Asyl und Migration. Country Report: Regular procedure. Asylum Information Database 2020. Available from: https://asylumineurope.org/reports/country/germany/asylum-procedure/procedures/regular-procedure/. Accessed 20 May 2021.

[30] Federal Office for Migration and Refugees. Studying in Germany. BAMF; 2020. Available from: https://www.bamf.de/EN/Themen/MigrationAufenthalt/ZuwandererDrittstaaten/Bildung/Studium/studium-node.html. Accessed 20 May 2021.

[31] Alberer M, Wendeborn M, Löscher T, Seilmaier M (2016). Erkrankungen bei Flüchtligen und Asylbewerbern (Illnesses among refugees and asylum seekers). Dtsch Med Wochenschr.

[32] Beermann S, Rexroth U, Kirchner M, Kühne A, Vygen S, Gilsdorf A (2015). Asylsuchende und gesundheit in Deutschland: Überblick über epidemiologisch relevante Infektionskrankheiten (asylum seekers and their health conditions in Germany: overview of epidemiologically relevant infectious diseases). Deutsches Ärzteblatt.

[33] Renner A, Hoffmann R, Nagl M, Roehr S, Jung F, Grochtdreis T, König HH, Riedel-Heller S, Kersting A (2020). Syrian refugees in Germany: perspectives on mental health and coping strategies. J Psychosom Res.

[34] Georgiadou E, Zbidat A, Schmitt GM, Erim Y (2018). Prevalence of mental distress among Syrian refugees with residence permission in Germany: a registry-based study. Front Psychiatry.

[35] Zbidat A, Georgiadou E, Borho A, Erim Y, Morawa E (2020). The perceptions of trauma, complaints, somatization, and coping strategies among Syrian Refugees in Germany-a qualitative study of an at-risk population. Int J Environ Res Public Health.

[36] Kikhia S, Gharib G, Sauter A, Vincens NCL, Loss J (2021). Exploring how Syrian women manage their health after migration to Germany: results of a qualitative study. BMC Womens Health.

[37] Spura A, Kleinke M, Robra BP, Ladebeck N (2017). How do asylum seekers experience access to medical care?. Bundesgesundheitsblatt Gesundheitsforschung Gesundheitsschutz.

[38] Ritchie J, Lewis J. Qualitative research. A guide for social science students and researchers: SAGE Publications; 2010.

[39] Grossoehme D, Lipstein E (2016). Analyzing longitudinal qualitative data: the application of trajectory and recurrent cross-sectional approaches. BMC Res Notes.

[40] Winiarska A, UW CoMR (2017). Qualitative longitudinal research: application, potentials and and challenges in the context of migration research.

[41] Shirani F, Henwood K (2011). Continuity and change in a qualitative longitudinal study of fatherhood: relevance without responsibility. Int J Soc Res Methodol.

[42] Morse J, Barret M, Mayan M, Olson K, Spiers J (2002). Verification strategies for establishing reliability and validity in qualitative research. Int J Qual Methods.

[43] Cane J, O'Connor D, Michie S (2012). Validation of the theoretical domains framework for use in behaviour change and implementation research. Implement Sci.

[44] French SD, Green SE, O'Connor DA, McKenzie JE, Francis JJ, Michie S (2012). Developing theory-informed behaviour change interventions to implement evidence into practice: a systematic approach using the theoretical domains framework. Implement Sci.

[45] Bojorquez I, Renteria D, Unikel C (2014). Trajectories of dietary change and the social context of migration: a qualitative study. Appetite..

[46] Glaser BG, Strauss AL (1999). Discovery of grounded theory. Strategies for qualitative research.

[47] Willing C, Willing C (2013). Grounded theory methodology. Introducing qualitative research in psychology.

[48] Mays N, Pope C (2000). Assessing quality in qualitative research. Br Med J.

[49] Mays N, Pope C (1995). Rigour and qualitative research. Br Med J.

[50] Mensink GB, Truthmann J, Rabenberg M, Heidemann C, Haftenberger M, Schienkiewitz A (2013). Obst- und Gemüsekonsum in Deutschland. Ergebnisse der Studie zur Gesundheit Erwachsener in Deutschland (DEGS1). Bundesgesundheitsblatt Gesundheitsforschung Gesundheitsschutz..

[51] Robert Koch Institut (2014). Daten und Fakten: Ergebnisse der Studie "Gesundheit in Deutschland aktuell 2012". Beiträge zur Gesundheitsberichterstattung des Bundes.

[52] Koch F, Heuer T, Krems C, Claupein E (2019). Meat consumers and non-meat consumers in Germany: a characterisation based on results of the German National Nutrition Survey II. J Nutr Sci.

[53] Mensink GB, Schienkiewitz A, Haftenberger M, Lampert T, Ziese T, Scheidt-Nave C (2013). Übergewicht und Adipositas in Deutschland. Ergebnisse der Studie zur Gesundheit Erwachsener in Deutschland (DEGS1). Bundesgesundheitsblatt Gesundheitsforschung Gesundheitsschutz..

[54] Scagliusi FB, Porreca FI, Ulian MD, de Morais SP, Unsain RF (2018). Representations of Syrian food by Syrian refugees in the city of Sao Paulo, Brazil: an ethnographic study. Appetite..

[55] Porreca FI, Unsain RAF, Carriero MR, Sato PM, Ulian MD, Scagliusi FB (2020). Dialogues and tensions in the eating habits of Syrian refugees living in Sao Paulo, Brazil. Ecol Food Nutr.

[56] Hunter-Adams J (2017). Exploring perceptions of the food environment amongst Congolese, Somalis and Zimbabweans living in Cape Town. Int Migr.

[57] Lecerof SS, Stafstrom M, Emmelin M, Westerling R, Ostergen PO (2017). Findings from a prospective cohort study evaluating the effects of international health Advisors' work on recently settled migrants' health. BMC Public Health.

[58] Renzaho AM, Halliday JA, Mellor D, Green J (2015). The healthy migrant families initiative: development of a culturally competent obesity prevention intervention for African migrants. BMC Public Health.

[59] Riedel J, Wiesmann U, Hannich HJ (2011). An integrative theoretical framework of acculturation and salutogenesis. Int Rev Psychiatry.

[60] Golchert J, Roehr S, Berg F, Grochtdreis T, Hoffmann R, Jung F, Nagl M, Plexnies A, Renner A, König HH, Kersting A, Riedel-Heller SG (2019). HELP@APP: development and evaluation of a self-help app for traumatized Syrian refugees in Germany - a study protocol of a randomized controlled trial. BMC Psychiatry.

